# A Patient with GOLD Stage 3 COPD « cured » by One-Way Endobronchial Valves

**DOI:** 10.3390/medicina55030065

**Published:** 2019-03-11

**Authors:** Eric Marchand, Jean-Paul d’Odemont, Michael V Dupont

**Affiliations:** 1 CHU-UCL-Namur, site Godinne, Université catholique de Louvain, Department of Pneumology, Institut de recherche expérimentale et Clinique (IREC), Av Dr Therasse 1, Yvoir, BE 5530, Belgium; 2 Laboratoire de Physiologie Respiratoire, URPhyM, NARILIS, Faculté de Médecine, UNamur. Rue de Bruxelles, 61, Namur, BE 5000, Belgium; 3 CHU-UCL-Namur, site Godinne, Université catholique de Louvain, Department of Pneumology, Av Dr Therasse 1, Yvoir - BELGIUM, BE 5530, Belgium; dod@skynet.be; 4 CHU-UCL-Namur, site Godinne, Department of Radiology, Av Dr Therasse 1, Yvoir , BE 5530, Belgium; michael.dupont@uclouvain.be

**Keywords:** COPD, treatment, lung hyperinflation, lung volume reduction, endobronchial valves

## Abstract

Lung hyperinflation is a main determinant of dyspnoea in patients with chronic obstructive pulmonary disease (COPD). Surgical or bronchoscopic lung volume reduction are the most efficient therapeutic approaches for reducing hyperinflation in selected patients with emphysema. We here report the case of a 69-year old woman with COPD (GOLD stage 3-D) referred for lung volume reduction. She complained of persistent disabling dyspnoea despite appropriate therapy. Chest imaging showed marked emphysema heterogeneity as well as severe hyperinflation of the right lower lobe. She was deemed to be a good candidate for bronchoscopic treatment with one-way endobronchial valves. In the absence of interlobar collateral ventilation, 2 endobronchial valves were placed in the right lower lobe under general anaesthesia. The improvement observed 1 and 3 months after the procedure was such that the patient no longer met the pulmonary function criteria for COPD. The benefit persisted after 3 years.

## 1. Introduction

Lung hyperinflation is a main determinant of dyspnoea, the most disabling symptom experienced by patients with chronic obstructive pulmonary disease (COPD) [1]. Lung hyperinflation associated with COPD usually has both a dynamic and a static component. Dynamic hyperinflation is secondary to expiratory flow limitation resulting from reduced airway calibre which results in an increased end-expiratory lung volume. It first occurs during physical exercise because of the increased ventilatory requirements but it can even be present at rest when the disease progresses. Dynamic hyperinflation adds to a higher equilibrium volume of the respiratory system (static lung hyperinflation) which results from the reduced lung compliance related to emphysema 

Accordingly, reducing lung hyperinflation is a major therapeutic goal for alleviating dyspnoea in patients with COPD. This is the main mechanism explaining the effect of inhaled bronchodilators which are the cornerstone of COPD maintenance therapy. Indeed, bronchodilators somewhat reduce lung hyperinflation by increasing maximal expiratory flows and dynamic hyperinflation. Their effect is however limited [1]. Accordingly, treatments aiming to reduce lung volume to a larger extent have been developed. By targeting emphysematous non-functional areas, lung volume reduction surgery was able to improve lung hyperinflation, dyspnoea and maximal expiratory flows in well selected emphysematous patients [2]. However, the enthusiasm around lung volume reduction surgery has been hampered by significant perioperative morbidity and mortality [3]. More recently, endobronchial techniques including one-way endobronchial valves (EBV) [4], coils [5], vapor [6] or sealant [7] have been developed with the hope to reduce periprocedural complications as compared to lung volume reduction surgery. These techniques aim to achieve the collapse of emphysematous lung areas in order to reduce lung volume. Among them, endobronchial valves (EBV) have been studied the most thoroughly. Their benefits have been demonstrated as compared to standard of care in several randomized clinical trials after 6 months [4,8,9,10,11] and more recently out to at least 12 months [12]. A positive impact on surrogates for improved survival (e.g., 6 min walk distance, BODE index and inspiratory capacity (IC)/total lung capacity (TLC) ratio) has also been demonstrated [13]. We here report the case of a woman with COPD and disabling symptoms who experienced a dramatic and sustained (e.g., 3 years) improvement after EBV treatment. The improvement was such that the patient no longer met the diagnostic criteria for COPD after treatment. To the best of our knowledge, such a dramatic improvement has never been reported in the literature.

The patient gave informed consent for the use of her personal data in the current report.

## 2. Case Report

A 69-year old woman was referred for possible lung volume reduction. She had a history of COPD with dyspnoea progressively worsening over 6 years. Her medical history included past smoking (stopped 17 years earlier and totalling 40 pack years), thyroidectomy for multinodular goitre, uncomplicated systemic hypertension and type 2 diabetes. At the time of referral, her treatment included inhaled tiotropium and salmeterol/fluticasone, levothyroxine, valsartan, hydrochlorothiazide and metformin.

She complained of dyspnoea grade 2–3 (modified Medical Research Council –mMRC-scale; that is, she stopped for breath after walking 200–300 meters (m) on the level) and acknowledged a sedentary lifestyle. She experienced less than one exacerbation per year. The COPD assessment test (CAT) score was 24/40, suggesting a high impact of COPD on the patient’s health and daily life.

Clinical examination showed severely diminished breath sounds at the lower part of the right hemithorax. Besides overweight (BMI 29.5 kg/m^2^), it was otherwise unremarkable.

As shown in Table 1, pulmonary function tests (PFT) showed severe airway obstruction (GOLD stage 3) with significant lung hyperinflation. Lung diffusion was relatively preserved. She walked 342 m on a 6-min walk test (6MWT) with oxygen saturation measured by pulse oximetry (SpO_2_) dropping from 97 to 92%. The BODE index was 5/10. According to the 2015 (time of the initial assessment in our centre) GOLD guidelines, she was classified as grade D for risk stratification (Grade B according to the current GOLD guidelines) [14].

**Figure 1:**
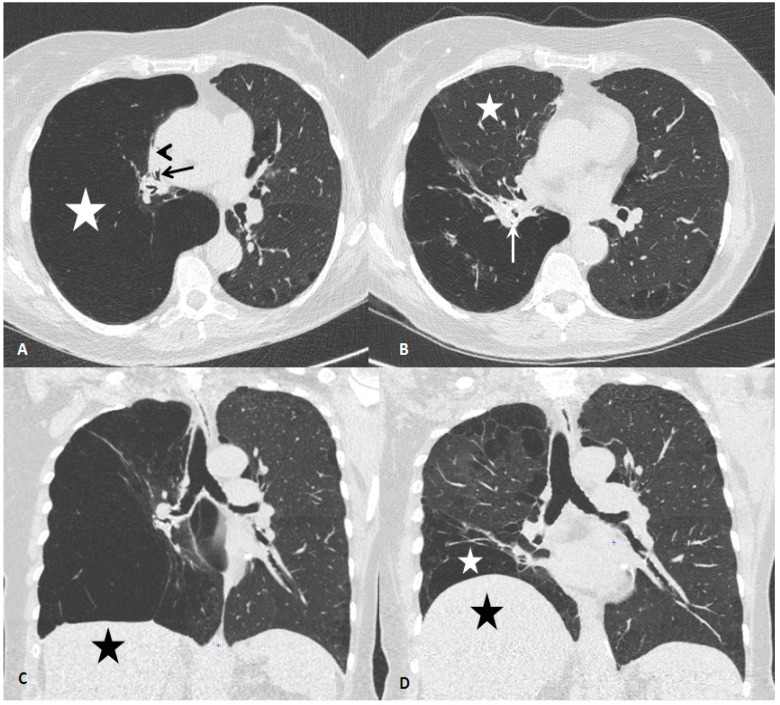
Chest computed tomography before and after endobronchial valves treatment. (**A**) Axial chest computed tomography (CT) at the level of the right middle bronchus (black arrow), before endobronchial valve treatment, showing complete atelectasis of the right middle lobe (black arrowhead) and extensive emphysema and hyperinflation of the right lower lobe (white star). (**B**) Axial CT at the same level 1 month after placement of endobronchial valves (EBV), showing right middle lobe re-expansion (white star). One valve is seen in the right lower lobe bronchus (white arrow). (**C**) CT coronal reconstruction before EBV treatment, showing markedly heterogeneous emphysema and diaphragm flattening (black star). (**D**) CT coronal reconstruction at the same level 1 month after EBV treatment, showing right lower lobe partial collapse (white star), right hemidiaphragm elevation (black star) and right- and backward mediastinal shift.

A chest X-ray showed right lung hyperinflation with a shift to the left of the mediastinum. A high-resolution computed chest tomography (HRCT) (Figure 1; panels A, C) showed mild paraseptal and centrilobular emphysema in both lungs with emphysematous destruction and severe hyperinflation of the right lower lobe. The latter was associated with contralateral mediastinal shift along with complete middle and partial right upper lobe atelectasis. Review of the chest CT performed 1 and 6 years earlier in another hospital showed that the right lower lobe experienced slowly progressive distension. Visual assessment of the HRCT suggested great fissure completeness.

A transthoracic echocardiography was unremarkable, without significant pulmonary hypertension (systolic pulmonary arterial pressure: 40 mmHg).

The patient was deemed to be a good candidate for EBV lung volume reduction and was first included in a pulmonary rehabilitation program. After 3 months of rehabilitation, dyspnoea was mildly improved (grade 2 mMRC) as was the CAT score (26/40). The 6 MWT was unchanged (340 m). Her chest auscultation and PFT were not significantly improved (Table 1).

After exclusion of collateral ventilation with the use of the Chartis Diagnostic System (PulmonX Intl, Neuchatel, Switzerland), as previously described [15], 2 one-way EBV (Zephyr; PulmonX Intl, Neuchatel, Switzerland; provided by RMS Medical Devices, Roosdaal, Belgium) were placed in the right lower lobe, under general anaesthesia. The post-procedural course was marked by fever 48 hours after valves placement. A chest X-ray showed ground glass opacities in the inferior part of the right lung while the right hemidiaphragm was shifted upwards. The patient was treated with amoxicillin-clavulanate. She rapidly improved and was discharged home on the 7th day without any change in inhaled therapy.

After one month, the patient reported marked improvement. She was no longer limited in her daily-life activities by dyspnoea (dyspnoea mMRC score 0–1). Her CAT score markedly improved (10/20) as did the 6 MWT (399 m). Chest auscultation still revealed diminished breath sounds on the posterior right side but asymmetry was reduced. HRCT showed a marked reduction in the right lower lobe volume (1233 mL versus 3491 mL before treatment) with accompanying right upper lobe re-expansion and disappearance of the mediastinal shift (Figure 1; panels B, D). 

The improvement in PFT was even more remarkable. Indeed, the patient no longer met the GOLD initiative spirometric criteria for COPD (Table 1) [14].

These improvements were confirmed at 4 months and were maintained at the latest control, nearly 3 years after the procedure. The SpO_2_ measured at rest were stable in the follow-up and the minimal SpO_2_ during the walk tests remained above 90%. Inhaled steroids were progressively tapered after treatment.

Despite the PFT and dyspnoea improvements, the patient experienced 5 exacerbations requiring ambulatory antibiotic treatment in the 3-year follow-up.

## 3. Discussion

Lung hyperinflation is a main determinant of dyspnoea, the most disabling symptom experienced by patients with COPD [1]. 

Among endobronchial lung volume reduction techniques, EBV have been the best studied. By inserting one or several valves to occlude either the lobar bronchus or all segmental bronchi of the target lobe, EBV treatment aims to induce lobar atelectasis. Several randomized trials clearly demonstrated significantly greater improvements in PFT and more importantly, in dyspnoea, quality of life and exercise tolerance with EBV as compared to standard of care. The mean reported improvement in FEV1 at 6 months in these trials ranged from 40 mL to 161 mL or 4 to 21% [4,8,9,10,11]. Similar improvements (e.g., mean FEV1: 106 mL) were reported in the only study out to at least 12 months [12]. The best results are however obtained when a collateral ventilation is excluded before EBV placement and when complete atelectasis of the target lobe is achieved [4,8,9,10,11,12]. Unsuccessful procedures generally result from the lack of atelectasis due either to technically unsuccessful treatment or the presence of interlobar collateral ventilation. The latter results in the persistence of ventilation of the target lobe through the interlobar collaterals, which impedes the atelectasis process after EBV placement [16]. The most common complication after EBV lung volume reduction is a pneumothorax, which is observed on the ipsilateral side in about 20% of treated patients. It can usually be managed with chest tube insertion. As it most commonly occurs after complete lobar atelectasis, it has no significant detrimental effect on the final clinical outcome [17].

When compared to the literature, the improvement observed in the present patient after EBV treatment was remarkable (710 mL or 80% increase in FEV1 3 years after the procedure). The patient no longer fulfilled the GOLD diagnostic criteria for COPD according to spirometry while she was classified as GOLD airway obstruction grade 3 prior to EBV treatment. Furthermore, the FEV1 and symptom improvements persisted largely unchanged after 3 years of follow-up. The changes in DL, CO are more difficult to interpret. There was a delayed improvement at 4 months in keeping with the changes in lung volumes but we observed a decline at 4 years. There were no clinical signs of anaemia at that time. More importantly, her symptoms were dramatically improved. This obviously does not mean that she was cured of her respiratory disease but highlights the potential of lung volume reduction treatment and the need for better criteria to identify patients who will benefit the most.

The pathophysiological basis for the PFT and symptoms improvements after lung volume reduction is more complex than a mere reduction in end-expiratory lung volume as nicely exemplified by the present case. Indeed, maximal expiratory flows are also improved as shown by the improvement in FEV1. It is generally accepted that improvements in maximal expiratory flows after lung volume reduction are due to increased lung elastic recoil [18]. However, several arguments also point to the role of the relief of a competition for space between distended emphysematous lung and healthier lung areas within a rigid chest wall. This allows for an improvement in vital capacity and accordingly in FEV1 and maximal expiratory flows, as elegantly analysed with a mathematical model [19,20]. The interplay with chest wall compliance may be substantial as suggested in an hamster model of emphysema. In this animal model, there was a lack of increase in maximal expiratory flows after surgical lung reduction, most probably owing to the high chest wall compliance in hamsters [21].

Predicting individual response to lung volume reduction remains a challenge. The heterogeneity of emphysema which makes it possible to target the most diseased lung areas for resection [22,23,24,25] has been shown to be an important predictor of improvement after surgical lung volume reduction. In that regard, the distribution of emphysema in the present case was very unusual. Emphysema was mostly distributed in the right lower target lobe, which was severely hyperinflated due to emphysema destruction. This certainly played a key role in the spectacular improvement after EBV treatment. Although positive results have been reported after EBV treatment in homogeneous emphysema [10], better results are to be expected in very heterogeneous emphysema and particularly lobar emphysema as exemplified by the present case. In clinical studies, only the RV/TLC ratio that was proposed in the mathematical model by Fessler and Permutt [19] was consistently shown to predict a positive functional outcome after surgical lung volume reduction [26,27,28]. Moreover, for EBV treatment, it has been shown that fissure integrity [29] and absence of collateral ventilation [16] are important for achieving lobar atelectasis.

Both a very heterogeneous emphysema distribution and a very high RV/TLC ratio with compression atelectasis and mediastinal shift on HRCT were indices predicting a favourable outcome after EBV treatment in the present case. In addition to the improvement in diaphragmatic function afforded by reduced hyperinflation, the relief of the compression of the healthier middle and upper right lobes largely accounted for the extraordinary improvement observed in this patient. This highlights the need for improvement in the selection criteria for bronchoscopic lung volume reduction in order to offer this technique to the patients expected to achieve the greatest functional response.

## 4. Conclusions

We here report the case of a COPD patient with markedly heterogeneous emphysema and severe lung hyperinflation who experienced long lasting dramatic improvements in both symptoms and PFT after EBV treatment. This case illustrates the major role of lung hyperinflation as a pathophysiologic mechanism explaining dyspnoea in COPD as well as the potential of lung volume reduction therapies which are restricted to very selected patients. 

## Figures and Tables

**Table 1:** Pulmonary function tests and other outcome measures before and after EBV treatment.

	Before PR	After PR	1 Month Post-EBV	4 Months Post-EBV	3 Years Post-EBV
Post-BD FEV1, L (% pred)	0.89 (45) *	0.85 (42)	1.67 (83)	1.62 (82)	1.56 (82)
Post-BD FVC, L (% pred)	1.36 (57) *	1.31 (54)	2.40 (99)	2.24 (94)	2.22 (96)
Post-BD FEV1/FVC	0.65*	0.65	0.70	0.72	0.70
RV, L (% pred)	5.60 (280)	5.19 (257)	3.36 (166)	3.47 (172)	4.10 (199)
TLC, L (% pred)	7.16 (150)	6.75 (141)	5.84 (121)	6.20 (130)	6.47 (137)
RV/TLC	0.78	0.77	0.57	0.56	0.63
DL, CO (% pred)	62	52	65	89	59
6 min walk distance (m)	342	340	399	428	412
CAT Score	27	23	10	9	12
Dyspnoea mMRC score	2–3	2	1	1	1
BODE index	4–5	4	0	0	0

*: As post-BD FEV1 and FVC were respectively 40 and 50 mL lower than pre-BD values, pre-BD values are reported. EBV: endobronchial valve(s); BD: bronchodilator; FEV1: forced expiratory volume in one second; L: litres; % pred: percent of the predicted value; FVC: forced vital capacity; RV: residual volume; TLC: total lung capacity; DLCO: CO transfer factor; m: meters; CAT: COPD assessment test; mMRC: modified Medical research Council; PR: pulmonary rehabilitation.
